# An innovative model for integrated delivery of prevention, screening and palliative care services of cancers at health and wellness centres in Assam

**DOI:** 10.3332/ecancer.2025.1983

**Published:** 2025-09-03

**Authors:** Rewati Raman Rahul, Nandini Vallath, Kunal Oswal, Ravikant Singh, Kumar Gaurav, Paul Sebastian, Venkataramanan Ramachandran, Arnie Purushottham

**Affiliations:** 1 Public Health, Karkinos Healthcare, Tata Cancer Care Foundation, Gaya 823001, India; 2 Department of Pain and Palliative Medicine, St. John’s Medical College & Hospital, Tata Cancer Care Foundation, Bengaluru 560034, Karnataka, India; 3 Screening and Early Detection, Karkinos Healthcare, Mumbai 400015, India; 4 Homi Bhabha Cancer Hospital and Research Centre, Doctors for You, Muzaffarpur 842004, India; 5 Independent, Tata Cancer Care Foundation, Dantewada 494449, India; 6 Clinical Governance and Early Detection, Karkinos Healthcare, Tata Cancer Care Foundation, Thiruvananthapuram 695017, India; 7 Karkinos Healthcare, Tata Trusts, Mumbai 400009, India; 8 King's Health Partners Comprehensive Cancer Centre Consultant Surgeon, Guy's and St Thomas' NHS Trust, Tata Cancer Care Foundation, London SE1 7EH, UK

**Keywords:** early detection, palliative care, cancer screening, palliative care, primary healthcare, non-communicable disease

## Abstract

There is a consensus on delivering prevention, early detection and palliative care services as effective cancer control strategies in primary healthcare settings; however, examples of practical application are few. The study describes the implementation of integrated delivery of preventive, early detection and palliative care needs assessment through the frontline healthcare workers at the Health and Wellness Centre. The study employed a master trainer team of dentist and nurses trained in prevention and needs assessment of palliative care services who would further provide the handhold training to the Community Health Officers (CHO), multi purpose workers and Accredited Social Health Activist for awareness, prevention and generalist palliative care needs assessment. 2106 households with 256 people were screened as a result, with an average of around 30 screenings a day. Screen positivity rates was found to be 3.1% for the oral cancer, for breast cancer it was 1.8% while for cervical cancer it was 3.4%. While 0.5% households were identified in need of palliative care, all screened positive cases were provided counselling for further diagnostics and care at the cancer centre in the district. The ambulance services of 102 available in the state were arranged for people willing to undergo the diagnostics. The evidence generated has the potential for practical application with further testing and strengthening in the field.

## Background 

The annual incidence of cancer in India for 2022 was 1.41 million and is expected to reach 1.75 million by the year 2030 [1], while the cancer-related deaths were 0.91 million in 2022 and are estimated to reach 1.15 million by the year 2030 [2].

The overall burden due to the high incidence of cancer is worsened due to the late presentation of patients to healthcare facilities. Of the patients seeking care from cancer care institutions, more than 60 percent of cases were reported in advanced stages, not amenable to curative treatment [3]. The lack of availability of standard facilities with skilled human resources closer to home results in patients travelling to far-off regions seeking treatment and care. This leads to high out-of-pocket expenses and dropout rates across the treatment pathway [4].

Government of India (GOI) adopted a policy in the year 2010 to implement breast, cervical and oral cancer screening with clinical breast examination (CBE), visual inspection with acetic acid (VIA) and oral visual inspection, respectively, through the National Programme for Prevention and Control of Cancer, Diabetes, Cardiovascular Diseases and Stroke (NPCDCS) [5].

The program was scaled gradually across the country with a defined operational guideline for the management of cancer. However, there were several implementation challenges and variability across different states. In 2023, GOI launched revised operational guidelines of the programme (National Programme for Prevention and Control of Non-Communicable Diseases: NP-NCD 2023-30) having focus on primary and secondary prevention, clinical support for non-communicable diseases (NCDs) and programme management with an objective to integrate NCD care at various healthcare delivery levels mainly focussing on health and wellness centre or Ayushman Arogya Mandir (AAM) [6].

Under the flagship health programme of Ayushmann Bharat, the GoI has identified the gap and developed a package of 12 service categories to be delivered under Comprehensive Primary Health Care, at the Health & Wellness Centres (HWC) commonly known as AAM. This package contains the screening of NCDs and the palliative care services [7].

The three most common cancers in India – oral, breast and uterine cervix cancer have well‑developed interventions for screening and early detection. They also have detectable early stages (mainly precancerous – oral and cervical) that are amenable to secondary prevention [8]. There is a consensus that about 60% of cancer deaths in India can be prevented with improved preventive and screening facilities alone. Moreover, generalist palliative care skills can help relieve the health-related suffering seen in different stages of the disease [9]. The frontline health care workers can be easily trained to achieve the required skills for these interventions. Building such capacity closer to patients in need can positively impact the cancer burden in the community. The role of frontline workers, including Accredited Social Health Activists (ASHAs), has already been demonstrated to be useful in the screening programmes [10]. It is hypothesised that frontline workers could play an important role in effective cancer control by integrated primary healthcare delivery of the screening of the three common cancers, as well as identification, assessment and referral of patients in the advanced stage of the disease, who need palliative care in the community [10, 11]. However, there has been very limited translation of this idea into practice in the community.

The current study tries to present this novel model of delivering integrated services of prevention, early detection and palliative care (PEP model) through the HWCs by training frontline health workers.

## Materials and Methods

**Figure 1.**
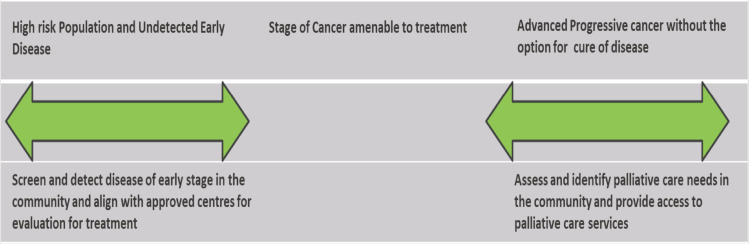
Shows the primary healthcare interventions planned across prevention, early detection and palliative care services for cancer care in the outreach. The green arrows are indicative of the two divergent ends of the cancer care conducted as a part of primary healthcare services.

The study was devised with an aim to assess the feasibility of conducting traditionally divergent activities like NCD screening and palliative care needs assessment in an integrated approach. Figure 1, named Primary Healthcare Interventions Across Prevention, Early Detection and Palliative Care Across Cancer Care Spectrum, demonstrates the intervention planned in the outreach.

The objectives of the study were

1. Assessment of palliative care needs in the community through a master trainer team equipped in the prevention and early detection of NCDs in the community.
2. Delivering NCD screening and primary palliative care services through frontline healthcare workers at the HWC.

### Programme design

**Figure 2.**
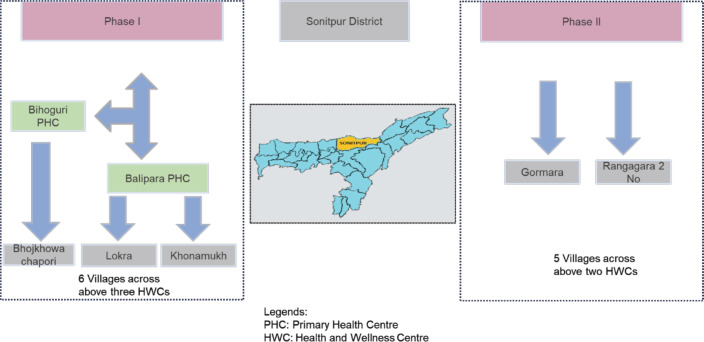
Is a representative depiction of the districts in the map of Assam and the public health facilities where interventions and data collection occurred during the study. The green colour in the boxes is the primary health centres, whereas the one in the grey inside the boxes shows the sub-centre health and wellness centres covered.

A MoU was signed by Tata Trusts, a Non-Governmental Organisation (NGO), with the Government of Assam for the effective implementation of the project. Some of the major provisions of the MoU were to a) design and implement a cancer control programme for prevention, early diagnosis, clinical down-staging, improved treatment, survival and cure rates and better palliative care, b) integrate and upgrade comprehensive cancer-related infrastructure, personnel and services across the state from the primary health centre to tertiary referral cancer hospitals and c) devise a cancer control programme that is innovative, evidence-based, locally appropriate, low cost and cost-effective. Sonitpur district was chosen due to the necessary administrative approval from the government, ongoing collaborative team activities and access to cancer care and palliative care for screen-positive cases. Figure 2, Intervention District and Health Facilities, shows the selection of the health facilities in the districts.

**Figure 3.**
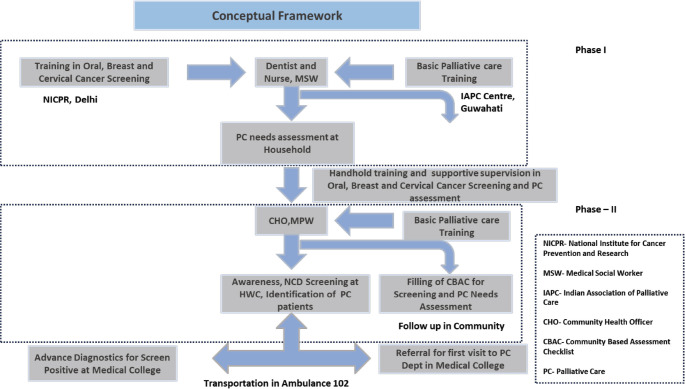
Shows the conceptual framework for the intervention and the referral pathways for the study. It describes the human resources, their type of training and referral pathway planned for the screen-positive cases during the study.

**Figure 4.**
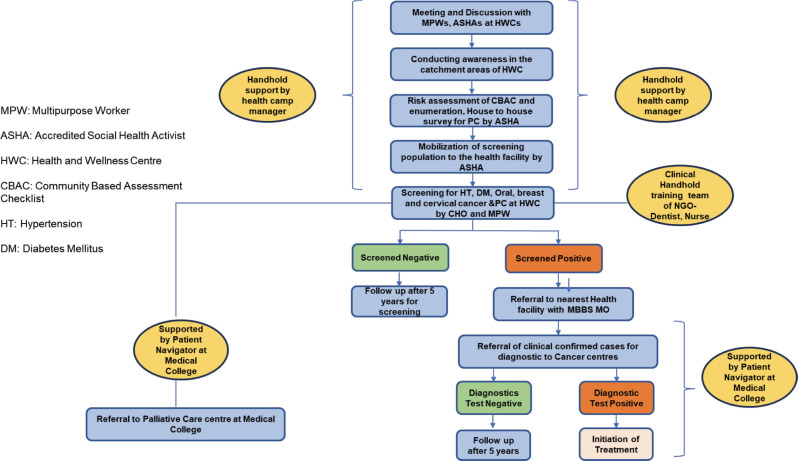
Is representative of the sequence of activities that were undertaken for the implementation of the study. The orange boxes show the type of human resources supervising and conducting the outreach activities. The red boxes show the negative outcome during screening and referral, whereas the green boxes show a favourable outcome during the implementation. The arrows and blue boxes are indicative of the sequence of events.

A two-phase project was devised to achieve the objectives. A four-member team of a dentist, a nurse, a medical social worker and a data entry operator was constituted. The dentist and the nurses were trained in screening for NCDs, including common cancers (oral, breast and cervical) at the National Institute for Cancer Prevention and Research. The same team underwent generalist palliative care training in an essential certificate programme conducted by the Indian Association of Palliative Care at the nearby centre in Guwahati. Appendix 1, named Training Schedule for Cancer Prevention, Early Detection and Palliative Care for Dentists and Nurses of the supplementary is attached for the training curriculum and schedule. While In the first phase, this team primarily conducted the needs assessment for palliative care at the HWC with the support of the frontline health workers using the Palliative care questionnaire integrated into the Community based assessment checklist shared in Appendix 2, in the second phase it played the role of master trainer and provided hands-on training to the frontline health workers (Community Health Officers, Multipurpose Health Workers and the ASHAs) to conduct awareness and screening for NCDs including the three common cancers (oral, breast and cervical) and facilitate assessment of palliative care needs by training the ASHAs on filling the Community Based Assessment Checklist (CBAC) forms. Linkages with the specialist department at the cancer centre and medical college were set up, and the team also helped develop a pathway for referral of the screen-positive cases arising from the screening for further diagnostic tests for confirmation of cancer. The conceptual framework and referral pathway for the Prevention, Early Detection and Palliative care (PEP) study, as well as the implementation of the PEP interventions, are shown in Figure 3 and Figure 4, respectively, with the same names.

Summary of the PEP activities planned at different levels of public healthcare is shown below.

### Household-level activities (conducted by ASHA)

- Mapping of the households for the eligible population for NCD screening.
- Filling out the CBAC forms for assessment of NCD screening and the palliative care needs assessment form.
- Dissemination of information regarding screening camps at HWC.

### Community-level intervention (conducted by ASHA)

- Facilitating awareness in the community through community camps regarding the
- Prevention of cancer and palliative care
- Mobilisation of the people to the HWC for screening camps.

### Health facility (CHO and MPW)

- Conduct screening camps at the HWC by CHO and MPW at HWC. Identifying people in need of care for palliative care through screening of CBAC forms submitted by ASHA. Manage referrals through arranged transportation for palliative care and NCD-screened cases.
- Follow-up of the screened positive and palliative care cases for generalist care.

## Results

**Figure 5.**
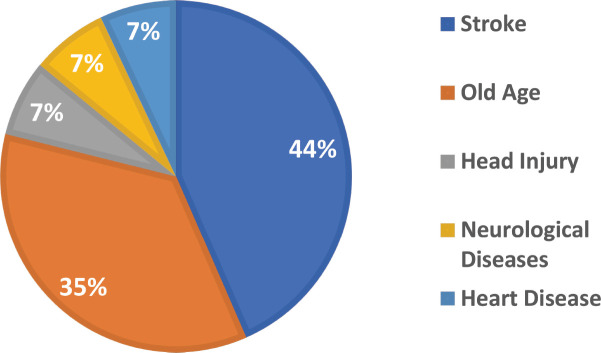
Shows the percentage of the types of diseases requiring palliative care during the needs assessment in phase I of the project. The data shows that the highest percentage of patients requiring palliative care is composed of stroke patients (44%) depicted in deep blue, followed by old age. (35%) depicted in orange. While head injury, neurological and heart patients contributed equally (7%), depicted in grey, yellow and light blue, respectively.

Results of the study are compiled in Table 1 below. During phase I of the needs assessment for palliative care, a total of 14 cases were identified to have palliative care needs across 1,035 households surveyed, with an average of 1.35% of households requiring palliative care. Figure 5 below shows the results of the data for the Needs assessment conducted for Palliative Care by disease in the first phase of the project. Stroke and old age-related illness were the top two contributors, with almost 44% and 35%, whereas heart, neurological and head injuries were other contributing factors.

In the second phase, a total of 2,106 households were covered, targeting a population of 11,811. A total of 11 talks and community awareness meetings were organised by the frontline health workers during the study period. 9 camps were organised in which a total of 256 people attended (Male: 63 and Female: 193) and were screened for oral cancer through oral visual examination methods. 163 females out of the total 193 participating females were eligible for breast cancer screening and were screened through the clinical breast examination method. 59 females out of the total 193 participating females were eligible for cervical cancer screening and were screened through visual inspection with the acetic acid method. Screen positivity rates were found to be 3.1% for oral cancer (in males, 4.76% and females 2.59%), for breast cancer, it was 1.8%, while for cervical cancer, it was 3.4%. All cancer-positive cases were counselled and followed up for their confirmatory diagnosis, in which 75% of oral suspected cases (66.6% male and 80% female) were reached and did their confirmatory test at referred centres, and 100% of breast and cervical cancer suspected cases did their confirmatory diagnosis. None of the screen-positive cases of oral, breast and cervical cancer were found to be positive after their confirmatory test at the higher centre. In the second phase of screening conducted for palliative care by the public health workers, 0.5% households required palliative care. All of these were provided services through the palliative care physician from the Baptist Christian Hospital and follow up done through the nurse-led home care team of the hospital.

## Discussion

Cancer screening in India has been a least priority due to multilevel barriers in implementation and is highly variable across 28 states and 8 union territories [12]. Community-based cancer screening programs in India have shown promise for early detection and improved outcomes. VIA has been widely used for cervical cancer screening, with sensitivity ranging from 16.6% to 82.6% and specificity from 82.1% to 96.8% [13]. Reports from the experiences from Tamil Nadu, the only state in the country to have scaled up services for the prevention of common cancers and other NCDs, have revealed that effective translation of evidence depends on a range of implementation issues, including context-specific communication strategies, provision of high quality services, linkages between screening, diagnosis and treatment and uptake of evidence by policy-makers and health care providers [14]. Engaging local health workers, such as Anganwadi workers, has improved attendance and follow-up rates in screening camps [15]. A large-scale breast cancer screening program in Kerala demonstrated the feasibility of door-to-door screening, with 93% of eligible women screened and 61% of detected cases in early stages [16]. Data from our study shows that the participation rate for oral, breast and cervical screening amongst those invited in community clinics were 100%. 84.5% and 30%, respectively. In a similar programme conducted in Jharkhand, the results for participation rates for oral, breast and cervical screening were 34.9%, 34.7%and 29.9% in an all women group [17], while in another study in Uttar Pradesh, this rate was 32% for cervical [18]. Major success of the programme as shown in the study, was a compliance rate of 75%, 100% and 100% amongst the screen positives of oral, breast and cervical cancer for confirmatory diagnosis within the public health system. The same was reported to be 14.6%, 48.1% and 28.3% community screening programme in Jharkhand [17] and 70% in a study from Kolkata [19] and 60% from a study in Kerala [16] from studies. However, the unique aspect of the implementation in this study was delivery of integrated programmes of cancer awareness, screening and palliative care services. Integrating palliative care into overall cancer control strategies like cancer prevention, early detection and curative treatments allows for a seamless response while making the best use of scarce resources [20]. The current model of PEP is timely and establishes a cost-effective and sustainable way for the government to provide an effective cancer control strategy at the primary level of care by effectively implementing the National Programme of NCD for Prevention and Control of Cancer, Diabetes, Cardiovascular Diseases and Stroke (NPCDCS) and National Program for Palliative Care at the HWC.

Experience at HWCs in Assam suggests that reducing the cancer burden in the community, through an integrated delivery as in the PEP approach, is a sustainable solution at primary-level public health facilities. It not only adds value to the public health programme by early detection and control of cancer, but is also effective in providing care to the incurable patients suffering from NCDs in the community near their homes.

Utilising their strengths, the NGO and government came together in making the initial effort at capacity building and stabilising the programme and shared the resources required for infrastructure, human resources and training as summarised in Table 2 below. This kind of synergistic partnership to work towards a shared vision of cancer control not only resulted in a demonstrated increase in the confidence of the primary-level healthcare providers but also led to an effective and judicious use of the resources. All of the above-mentioned factors resulted in better outcomes of the health programmes in the community.

## Limitations

The study shows data from the very early stages of the program with intervention conducted over a small number of health facilities in a district. There is a need for generating further evidence from other geographies in a diverse setting. While the possibility of delivering primary care for PEP activities is possible, there will be a constant need for handholding and supervision before the services are regularly delivered at the HWCs. With further refresher training and monitoring within the health systems, the participation rates for the screening programme are more likely to go up. Presence of a nodal centre with well-established preventive and palliative care departments is an essential prerequisite for providing diagnostics and curative services for screen-positive and palliative care services. This project has already established cancer care and palliative care centres in the districts before the community training of the frontline health workers was planned. However, this may not be feasible for all the districts and would require setting up such departments first as nodal centres.

## Conflict of interest

None of the authors have declared conflict of interest.

## Ethical Declaration

Necessary consents were taken from the participants during the course of screening and the same has been attached at the end of the documents.

## Author contributions

Conception and design: Nandini Vallath, Rewati Raman Rahul, Kunal Oswal

Administrative support: Ramachandran Venkataramanan, Kunal Oswal

Collection and assembly of data: Ravikant Singh, Kumar Gaurav, Rewati Raman Rahul

Data analysis and interpretation: Rewati Raman Rahul, Nandini Vallath, Kunal Oswal

Technical inputs and manuscript review: Nandini Vallath, Kunal Oswal, Paul Sebastian, Arnie Purushotham

Proof reading: Kunal Oswal, Paul Sebastian

Manuscript writing: All authors

Final approval of manuscript: All authors

Accountable for all aspects of the work: Rewati Raman Rahul, Kunal Oswal

## Figures and Tables

**Table 1.** Result of the PEP pilot conducted in Sonitpur.

Phase I. Palliative care needs assessment				
HWCs	Households surveyed	Patients identified with a need for palliative care		
		Male	Female	% of Total
Lokra, Balipara	586	2	6	1.36
Bhojkhowa Chapori	449	4	2	1.33
Total	1,035	6	8	1.35

Phase II. Prevention				
HWCs	Population	Awareness talks and community meetings organized		
		By ASHAs	By MPWs	Total
Gormara	5,439	3	2	5
Rangarao 2	6,372	3	3	6
Total	11,811	6	5	11

Phase II. Screening/Early detection															
HWCs	No of camps organized	Person screened for oral cancer			Screen positive for oral cancer						Compliance to referral				Total
					Male		Female				Male		Female		
		M	F	Total	*N*	%	*N*	% of Total	Total (*N*)	%	*N*	%	*N*	%	0
Gormara	5	44	109	153	2	4.5	2	1.8	4	2.6	1	50	2	100	0
Rangarao 2	4	19	84	103	1	5.2	3	3.5	4	3.8	1	100	2	66.6	0
Total	9	63	193	256	3	4.7	5	2.5	8	3.1	2	66.6	4	80	0

HWCs	No of camps organized	Female attended the camp	Female screened for breast cancer		Screened positive for breast cancer		Compliance to referral		Confirmation case of breast cancer
			*N*	%	*N*	%	*N*	%	
Gormara	5	109	94	86.2	2	2.1	2	100	0
Rangarao 2	4	84	69	82.1	1	1.4	2	100	0
Total	9	193	163	84.5	3	1.8	3	100	0

HWCs	No of camps organized	Female attended the camp	Female screened for cervical cancer		Screened positive for cervical cancer		Compliance to referral		Confirmation case of cervical cancer
			*N*	%	*N*	%	*N*	%	
Gormara	5	109	15	13.7	1	6.6	1	100	0
Rangarao 2	4	84	44	52.3	1	2.2	1	100	0
Total	9	193	59	30.6	2	3.4	2	100	0

Phase II. Palliative care.							
HWCs	Households surveyed	Patients in need of palliative care			% of Total in need of palliative care	Compliance to referral	
		Male	Female	Total		*N*	%
Gormara	1,142	1	3	4	0.3	4	100
Rangarao 2	964	4	3	7	0.7	7	100
Total	2,106	5	6	11	0.5	11	100

**Table 2.** Resources shared by the Tata Trusts and the State Government of Assam (NHM).

Resources	Tata trusts	NHM (Govt of Assam)
Infrastructure	Vehicle and fuel cost	HWC space, consumables, equipment, and medicines for screening
Human resources	Dentist, Nurse, medical social worker, and data entry operator	Health personnel of the HWC (ASHA, MPW and CHO)
Training		
• Training content	• IEC material for awareness and cancer awareness • IEC material for palliative care • Resource material for palliative	• Printed and distributed by NHM • Existing operational guidelines of the health programmes
• Master trainers	Prevention, early detection and palliative care	-
• Training cost and incentives	• TA, DA for the trainers • Honorarium for the trainers • Mobility support for the outreach team	• TA, DA of the HWC staff • Incentives to ASHA for filling CBAC forms

## APPENDIX FILE

### Appendix - 1. Training schedule for cancer prevention, early detection and palliative care for dentist and nurses.

**Table:**

Training schedule for dentists- cancer prevention and early detection	
**Topic**	**Duration**
**Day 1**	
Pre-test questionnaire	30 minutes
Screening and early detection of oral cancer	30 minutes
Methods of screening for oral Cancer (Videos and practice)	1 hour
CLINICS	3 hours
Epidemiology and etiology of oral cancer	1 hour
Debriefing	30 minutes
**Day 2**	
Recap of day 1	30 minutes
Oral potentially malignant disorders	1 hour
CLINICS	3 hours
Oral cancer-clinical features, signs and symptoms and diagnosis Biopsy techniques	1 hour
Debriefing	30 minutes
Day 3	
Recap of day 2	30 minutes
Harmful effects of tobacco	45 minutes
CLINICS	3 hours
Tobacco and areca nut ill effects and its addiction	45 minutes
Debriefing	30 minutes
Day 4	
Recap of day 3	30 minutes
Behavioral intervention for tobacco cessation	45 minutes
CLINICS	3 hours
Pharmacological intervention for tobacco cessation	30 minutes
Debriefing	30 minutes
Day 5	
Recap of day 4	30 minutes
Management of relapsed cases	30 minutes
CLINICS	3 hours
Role play on tobacco cessation	1 hour
Debriefing	30 minutes
Day 6	
Recap of day 5	30 minutes
Case presentation	30 minutes
CLINICS	3 hours
Setting up tobacco cessation services	1 hour
Debriefing	30 minutes
Day 7	
Recap of day 6	30 minutes
Role of health professionals in tobacco cessation and oral cancer screening	30 minutes
CLINICS	3 hours
Regulatory framework for tobacco control in India	45 minutes
Debriefing	30 minutes
Day 8	
Recap of day 7	30 minutes
CLINICS	3 hours
Trainee presentations	45 minutes
Debriefing	30 minutes
Day 9	
Recap of days1–8	1 hour
Trainee presentations	1 hour
CLINICS	3 hours
Assignment discussion	1 hour
Debriefing	30 minutes
Day 10	
Post-training knowledge assessment	1 hour
Post-training skill assessment using the checklist	1 hour 30 minutes
Certificate distribution	30 minutes
Feedback from the trainees	30 minutes

**Table:**

Training schedule for nurses in NCD prevention and early detection		
Day 1		
	**Topic**	**Duration**
Theory sessions	Pre-training knowledge assessment	15 minutes
	Goals and objectives of the training	30 minutes
	Introduction to Universal Screening of Common NCD	30 minutes
	Key tasks of Staff nurses in cancer prevention	30 minutes
	Rationale for screening, target age group; risk factors, signs and symptoms, etiopathogenesis of cervical/Breast/Oral cancer	45 minutes
	Natural history of cervical cancer	30 minutes
Practical sessions	Anatomy of female reproductive organs about cervix (using a model)	1 hour
	Per speculum examination using pelvic model	2 hours
	Summary of the day	30 minutes
Day 2		
Theory sessions	Recap of day 1	30 minutes
	HPV and cervical cancer	30 minutes
	Counselling for cervical cancer screening	30 minutes
	Video demonstration of per speculum examination to visualize the cervix	30 minutes
	Pictures of normal variants of cervix	30 minutes
	VIA procedure and principle of test	30 minutes
	Principles of VILI	15 minutes
	Confirmation of VIA positives with VILI	15 minutes
Practical sessions	Preparation of 5% acetic acid	30 minutes
	Demonstration of VIA on women by the trainer (observation of the procedure)	2 hours 30 minutes
	Summary of the day	30 minutes
Day 3		
	Recap of day 2	30 minutes
	Equipment, supplies, infection prevention items and preparing the woman	30 minutes
	Demonstration of preparing 0.5% Chlorine solution	30 minutes
	Practice in small groups: Counselling for cervical cancer screening	1 hour 30 minutes
	Supervised demonstration of VIA in the gynecology OPD, gynecology ward and cervical cancer screening clinic (Trainer guides the trainees one by one to carry out VIA test)	3 hours 30 minutes
	Summary of the day	30 minutes
Day 4		
	Recap of day 3 (trainee will be asked about the ease of the VIA procedure).	30 minutes
	Interpreting results of VIA	30 minutes
	Documenting results of VIA	30 minutes
	Pictures of positive VIA test	30 minutes
	VIA positive, what next?	30 minutes
	Skills practice in the classroom • Counseling for screening for cervical cancer • Getting consent from women undergoing the test Screening cervix with VIA	1 hour
	Hands-on demonstration of VIA in women (done by trainees under supervision)	3 hours
	Summary of the day	30 minutes
Day 5		
	Recap of day 4	30 minutes
	Overview of the “Screen and Treat” approach	30 minutes
	Demonstration of cryotherapy /Thermal ablation on model (apple, potato etc.)	1 hour
	Side effects of cryotherapy /Thermal ablation procedure	30 minutes
	Pre-and post-procedure counselling Post advice/instructions	30 minutes
	Independent practice of VIA test on women	3 hours
	Summary of the day	30 minutes
Day 6		
	Recap of day 5	30 minutes
	Supervised practice of performing per speculum examination and VIA on model using a checklist	2 hours
	Slideshow of positive VIA and VILI pictures	30 minutes
	Anatomy of female breast	30 minutes
	Overview of breast cancer screening	30 minutes
	Clinical breast examination (CBE): Demonstration of the model	1 hour
	Hands-on VIA/VILI/CBE	3 hours
	Summary of the day	30 minutes
Day 7		
	Recap of day 6	30 minutes
	Overview of oral cancer screening	30minutes
	Oral visual examination (OVE) Demonstration on patients	45 minutes
	OVE hands-on	2 hours
	Oral potential malignant disease (OPMD) Slide show of lesions	30 minutes
	Slide show of lesions	30 minutes
	Tobacco cessation	30 minutes
	Tobacco cessation (Role play by trainees)	30 minutes
	OVE by trainees under supervision	1 hour 30 minutes
	Summary of the day	30 minutes
Day 8		
	Recap of day 7	30 minutes
	Hands-on VIA/VILI /OVE/CBE on patients	3 hours
	Sterilization of instruments used during screening	45 minutes
	Tobacco cessation- hands-on with patients	1 hour
	Summary of the day	30 minutes
Day 9		
	Recap of days1–8	1 hour
	Addressing/answering women’s concerns regarding cervical/Breast/oral cancer screening (Role play by trainees)	2 hours
	Hands-on VIA /VILI/OVE/CBE /Tobacco cessation on patients	3 hours 30 minutes
	Summary of the day	30 minutes
Day 10		
	Post-training knowledge assessment	1 hour
	Post-training skill assessment using the checklist	1 hour 30 minutes
	Certificate distribution	30 minutes
	Feedback from the trainees	30 minutes
Common palliative care training curriculum for dentists and nurses in palliative conducted at state cancer institute, Guwahati (IAPC Centre)		
Part-A		Part-B
• Online half-day theory weekend classes • Virtual submission of a reflective case history evaluated by National Faculty. • Online written examination on theory classes		• A 10 day of hands-on training in palliative care at a IAPC authorised course centre with a mentor
Content • Introduction to palliative care • Communication skills • Spirituality, ethics in medical practice, psychological issues • Management of pain and other symptoms • Nursing issues • Palliative care in HIV/AIDS • Care of the elderly and paediatric palliative care • Palliative care emergencies • End of life care		

### Appendix 2. Palliative care questionnaire for ASHA workers.

**Table:**

General information	
Name of ASHA	Village/Ward
Name of MPW/ANM	Sub Centre (in case of rural areas)
PHC/UPHC	Date
Individual details	
Name	Any identifier (Aadhar Card, UID, Voter ID, other)
Age	State Health Insurance Scheme: (Y/N)
Sex	Telephone No.
Address	

**Table:** Part A: Risk assessment

Question	Range		Circle any	Write score
1. What is your age? (in complete years)	30–39 years		0	
	40–49 years		1	
	≥ 50 years		2	
2. Do you smoke or consume smokeless products such as gutka or khaini?	Never		0	
	Used to consume in the past/ Sometimes now		1	
	Daily		2	
3. Do you consume alcohol daily	No		0	
	Yes		1	
4. Measurement of waist (in cm)	Female	Male		
	80 cm or less	90 cm or less	0	
	81–90 cm	91–100 cm	1	
	More than 90 cm	More than 100 cm	2	
5. Do you undertake any physical activities for a minimum of 150 minutes in a week?	At least 150 minutes in a week		0	
	Less than 150 minutes in a week		1	
6. Do you have a family history (any one of your parents or siblings) of high blood pressure, diabetes and heart disease?	No		0	
	Yes		2	
Total score				

A score above 4 indicates that the person may be at risk for these NCDs and needs to be prioritised for attending the weekly NCD day

**Table:** Action: Ask if the patient has any of these symptoms

B1: Women and Men	Yes/No	B2: Women only	Yes/No
Any one in family currently suffering from TB*		Lump in the breast	
Are you currently taking anti-TB drugs*		Blood-stained discharge from the nipple	
History of TB **		Change in shape and size of breast.	
Shortness of breath		Bleeding between periods	
Coughing for more than 2 weeks**		Bleeding after menopause	
Blood in sputum**		Bleeding after intercourse	
Fever for > 2 weeks**		Foul-smelling vaginal discharge	
Loss of weight**			
Night sweats**			
History of fits			
Difficulty in opening mouth			
Ulcers/patch/growth that has not healed in 2 weeks			
Any change in the tone of your voice			
In case of individual answers Yes to any one of the above-mentioned symptoms, refer the patient immediately to the nearest facility where a Medical Officer is available.			

* If the answer is yes, tracing of all family members is to be done by ANM/MPW

** If the response is Yes- action suggested: Sputum sample collection and transport to the nearest TB testing centre

**Table:** Part C. Circle all that apply

Type of fuel used for cooking – Firewood/Crop Residue/ Cow dung cake/Coal/Kerosene
Occupational exposure – Crop residue burning/burning of garbage – leaves/working in industries with smoke, gas and dust exposure such as brick kilns and glass factories etc.

**Table:** Part D – Palliative care assessment Questionnaire by the ASHA to identify those needing palliative care in the family/community

Screening questions	Response	
	Yes	No
Is anyone in your family, suffering from an illness for more than 3 months?		
Is anyone in your family, unable to do their activities of daily living for more than 3 months?		
Is anyone in your family, unable to go for regular work for more than 3 months?		
Is anyone in your family, mostly bed-bound due for more than 3 months?		

If the answer is YES to any of the questions above, please note the following. Name of the patient:												
**Gender**		Female	Male		**Age**			**Caregiver**				
**Is she/he suffering from any of the following problems?**												
O	Breathlessness		O	Pain		O	Immobility		O	Vomiting		
O	Fever		O	Unconscious		O	Loose motion		O	Constipation		
O	Wound		O	Bleeding		O	Cough		O	Swelling		
O	Swallow difficult		O	Fits		O	Old-age issues		O	Confusion		
**Is she/he suffering from any of the following illnesses?**												
O	Heart disease		O	Lung disease		O	Cancer		O	Kidney disease		
O	HIV/AIDS		O	Persistent pain		O	Mental illness		O	Brain/nerve illness		
O	Other		O	Joint disease		O	Memory loss		O	Disease since birth		
**Where do you now go for medical help?**												
O	HWC/ ASHA		O	PHC		O	CHC		O	District hospital		
O	Medical College		O	Private hospital		O	Ayurvedic / Homeopathic doctor		O	Local healer		
O	Nowhere to go											
